# The ethics and editorial challenges of internet-based research

**DOI:** 10.1186/s12916-014-0124-3

**Published:** 2014-07-15

**Authors:** Stephanie Harriman, Jigisha Patel

**Affiliations:** 1 BioMed Central, Floor 6, 236 Gray’s Inn Road, London WC1X 8HB, UK

**Keywords:** Internet-based research, Ethics, Ethics approval, Informed consent

## Abstract

The internet has opened up vast possibilities for research. An increasing number of studies are being conducted using the internet as both a source of data and a venue for research. Use of the internet in research has created many challenges, not just for those conducting and reviewing the studies, but also for editors publishing this work. Two key issues raised by internet-based research are ethics approval and informed consent.

While some guidance exists regarding the ethics and consent of internet-based research, and some institutions provide their own guidelines, there appears to be a lack of definitive national standards.

We discuss the issues surrounding ethics and consent for internet-based research and the need for a consensus on how to address these issues to ensure consistency.

## Background

Facebook is a well-known online social networking site that, by the end of 2013, was reported to have 1.23 billion users worldwide [[1]]. Despite its main purpose as a social networking site, data posted on Facebook was recently used in a large scale research project, causing public outcry [[2]]. The research investigated whether emotional states can be transferred to others through social contagion. By reducing the amount of negative or positive content visible in a user’s News Feed, the researchers were able to investigate whether this had an effect on those users’ own posting behaviours [[3]].

The outcry surrounding the publication of this research has brought the ethical issues of internet-based research to the forefront. While Facebook’s study appears unique in size and controversy, internet-based research involving human subjects is not new. It is an issue that we, as journal editors, have been dealing with for some time.

We have encountered many studies that involve the use of the internet in some way. We have seen analyses of data posted on online fora, analyses of videos posted on YouTube and studies looking at the effect of website content on health-related behaviours. All of these studies have raised questions, including whether the protocol used required approval from an ethics committee, whether informed consent was obtained, and the extent to which participants knew that their data might be used for research.

## What is internet-based research?

Studies may now involve participants across the world. This allows for larger sample sizes, as demonstrated by the recent Facebook study, which involved 689,003 people [[3]].

“Internet-based research” is a broad term and includes many different study designs of varying levels of invasiveness and risk. Internet-based research can include both “the Internet as a *tool for research*” [[4]] and “the Internet as a *locale or venue for research*” [[4]]. It encompasses: the use of information already available on the internet (with no direct interaction with human subjects); using the internet as an interventional tool; using the internet to recruit research subjects; research about the internet and its effects; and research about internet users [[4]].

The possibilities offered by the internet bring with them many challenges, including verification of participant identity, follow-up and support of participants, ethics approval, informed consent and support of vulnerable groups. In this editorial we focus on what we see as the two main issues for editors – ethics and informed consent.

## Who is responsible for ethics approval of internet-based research?

Journals require that research involving human subjects, their data or material must have been performed in accordance with the Declaration of Helsinki and approved by an appropriate ethics committee. This also applies to internet-based research. An appropriate ethics committee is usually accepted to be an ethics committee *local* to the participants, and therefore able to weigh up the risks and benefits to *local* participants. Where research takes place in a single or few locations, this is straightforward, but how does this work for internet-based research? There is no ‘local’ ethics committee and researcher may not even know the locations of participants. Does an ethics committee in one country have jurisdiction to grant approval of a study that involves participants in another country? Should ethics approval be sought in every country from which participants may take part? Perhaps even more challenging is when researchers are based in a country where ethics approval is not required for the particular study design, but participants may be based in a country where it is. These questions raise ethical and logistical challenges which must be balanced against each other.

## What are the issues surrounding informed consent for internet-based research?

A fundamental principle underpinning research involving human subjects is the need for informed consent. The need for informed consent from research participants is stated in the Declaration of Helsinki [[5]] and reflected in our editorial policies [[6]].

The Facebook study states that, as all users agree to their Data Use Policy prior to creating an account, they have given informed consent to participate in this research [[3]]. Facebook’s Data Use Policy states that they may use the information about their users *“for internal operations, including troubleshooting, data analysis, testing, research and service improvement”* [[7]]. It is our interpretation that this does not include research for external publication. It is also not possible to guarantee that those posting were aware of this statement, and therefore that their data could be used in scientific research.

Internet-based research also raises issues surrounding data protection and participant privacy. This is particularly a problem when data that was posted on the internet for other purposes is then used for research. The assumption is that, if information is posted on the internet and in the public domain, it can be considered available for use for any purpose [[4]]; but how far are participants aware of this assumption when they post their data? This becomes particularly problematic if research subjects are children or other vulnerable groups who may post information about themselves without understanding the extent to which their information could be disseminated or used. While individual countries have regulations, such as the Data Protection Act in the UK [[8]], that control the use of private data, there needs to be a global consensus on the use of publically accessible data when the extent of the owners’ (the study participants’) awareness of its use is unclear, especially when the participants are from a country that is different from the researchers’ or are a vulnerable group.

## Existing guidance

We are not aware of any definitive national guidance or legislation specifically concerning internet-based research. While some guidance does exist, it is not consistent or comprehensive. Some individual institutions, both in the UK and USA, provide guidance for their researchers. The key points of a selection of this guidance are summarised in Table 1 for illustrative purposes.

**Table 1:** Examples of topics covered, and guidance on informed consent for internet-based research from selected UK and US academic institutions

**Institution**	**Topics covered in guidance**
University of Bedfordshire, UK	- Main focus on consent.
http://www.beds.ac.uk/research/iasr/ethics/onlineresearch [[9]]	- **Consent:** Online information may be freely quoted and analysed without consent if it is officially, publicly archived, not password protected and not prohibited by the site’s policy. For everything else, consent is required.
University of Brighton, UK http://about.brighton.ac.uk/hss/fregc/ [[10]]	- Covers privacy, anonymity, informed consent, potential harm or intrusion, access to participation and reliability of data.
	- Includes Association of Internet Researchers report.
	- **Consent:** highlights difficulties of obtaining informed consent and suggests possible approaches including retrospective consent after data collection with withdrawal of data from those not consenting, or seeking advice from website owner/administrator on the most appropriate way to obtain consent.
University of Connecticut, USA http://irb.uconn.edu/internet_research.html [[11]] and Penn State, USA http://www.research.psu.edu/policies/research-protections/irb/irb-guideline-10 [[12]]	- Cover recruitment, data collection, server administration, data storage/disposal and informed consent.
	- States that protocols for internet-based research are reviewed using the same standards and considerations as all other research activities.
	- **Consent:** Anonymous internet surveys may require only “I agree” or “I disagree” buttons, but IRB may require a written consent form to be mailed or faxed by participants. Researchers should not guarantee confidentiality or anonymity.
University of Cornell Office of Research Integrity and Assurance Institutional Review Board, USA http://www.irb.cornell.edu/faq/ [[13]]	- Focus on consent.
	- **Consent:** it may sometimes be appropriate to use implied informed consent for internet research, e.g. by completion of an online questionnaire. Participants should be informed that completion of questionnaire implies consent and should be shown consent information. In some cases an emailed “consent form” in lieu of a traditional paper informed consent form may be appropriate.

IRB; institutional review board.

In addition to this guidance from individual institutions, the Association of Internet Researchers has produced a report containing a series of considerations for researchers and those with oversight of internet-based research [[14]]. In the USA, the Secretary's Advisory Committee on Human Research Protections has produced a document outlining considerations and recommendations relating to conducting and reviewing internet-based research, which includes a list of twenty regulatory considerations [[4]].

For manuscripts reporting standard, non-internet research, our editorial polices aim to ensure the work we publish has been conducted ethically and within the required national regulations and frameworks. Given the lack of definitive and comprehensive guidance for internet-based research, we often struggle to know whether research conducted using the internet is ethical and conducted in compliance with the authors’ local regulations and laws such as the Data Protection Act in the UK.

## The need for policy on internet-based research

In light of the issues that we have experienced, and the apparent lack of definitive guidance, earlier this year we contacted the chairs of National Health Service (NHS) ethics committees (equivalent to institutional review boards (IRB) in the USA) in the UK to ask how they review proposals for internet-based research. We sought advice on four questions, listed in Table 2.

**Table 2:** Questions sent to chairs of UK NHS ethics committees

1.	Does your ethics committee have any formal guidance for researchers on the conduct of internet research?
2.	If you receive protocols for internet-based research, how do you assess issues of participant consent, especially for children/adolescents?
3.	How do you assess protocols that might include participants from outside of the ‘jurisdiction’ of your ethics committee, for example, if there could be participants or patients from other countries taking part?
4.	Do you make a distinction in requirements for consent between research on data already collected and available on the internet and prospective research that aims to collect data via the internet? If so, what is that distinction and how do you apply it in practice?

Of the 86 committees we contacted, we have to date received responses from three committees. They told us that they review research proposals that use the internet in the same way as other forms of research and that, so far, they have little experience of reviewing such research. This indicates that there is currently no formal guidance for the conduct of internet-based research in the UK.

## Conclusions

It is inevitable that the internet will increasingly become a ‘venue’ and source of data for human research. Guidance on the ethical conduct of such research is needed, not just for those who conduct and review the research, but for journal editors too. It is important that guidelines exist to ensure that internet-based research meets the same ethical and publication standards set for other research involving human subjects. A consensus on ‘best practice’ for internet-based research will be difficult to achieve given the very broad range of research circumstances in which it is used. We currently deal with these issues as they arise on a case by case basis and would welcome comments and views from fellow editors on what they would consider to be best practice.

## Competing interests

SLH and JP are both employees of BioMed Central and are Medical Editors of *BMC Medicine.*

## Author contribution

SLH and JP contributed to drafting this editorial.

## References

[B1] http://www.theguardian.com/technology/2014/feb/04/facebook-10-years-mark-zuckerberg Kiss J: **Facebook's 10th birthday: from college dorm to 1.23 billion users.** *Guardian*. .

[B2] http://www.theguardian.com/technology/2014/jun/29/facebook-users-emotions-news-feeds Booth R: **Facebook reveals news feed experiment to control emotions.** *Guardian*. .

[B3] Kramer A Guillory J Hancock J Experimental evidence of massive-scale emotional contagion through social networks PNAS 2014 111 8788 8790 10.1073/pnas.1320040111 24889601 PMC4066473

[B4] www.hhs.gov/ohrp/sachrp/commsec/attachmentbsecletter20.pdf Secretary's Advisory Committee on Human Research Protections: **Considerations and recommendations concerning internet research and human subjects research regulations, with revisions.** 2013. .

[B5] http://www.wma.net/en/30publications/10policies/b3/World Medical Association: **Declaration of Helsinki.**.

[B6] http://www.biomedcentral.com/about/editorialpolicies BioMed Central: **Editorial Policies.**.

[B7] https://www.facebook.com/about/privacy Facebook: **Data Use Policy.**.

[B8] http://www.legislation.gov.uk/ukpga/1998/29/contents **Data Protection Act 1998.**.

[B9] http://www.beds.ac.uk/research/iasr/ethics/onlineresearch University of Bedfordshire: **Ethical Guidelines for the Online Researcher.**.

[B10] http://about.brighton.ac.uk/hss/fregc/Ethical-internet.pdf University of Brighton: **Ethical issues for consideration when conducting internet research.**.

[B11] http://irb.uconn.edu/internet_research.html University of Connecticut: **Guidance for computer and internet-based research involving human subjects.**.

[B12] http://www.research.psu.edu/policies/research-protections/irb/irb-guideline-10 The Pennsylvania State University: **IRB Guideline X - Guidelines for Computer- and Internet-Based Research Involving Human Participants.**.

[B13] http://www.irb.cornell.edu/faq/Cornell University Office of Research Integrity and Assurance: **Frequently Asked Questions.**.

[B14] www.aoir.org/reports/ethics2.pdf **Association of Internet Researchers.**.

